# Bridging Gaps in Heart Failure Science: Toward a More Integrated Future

**DOI:** 10.3390/life15101613

**Published:** 2025-10-16

**Authors:** Ju-Chi Liu, Tzu-Hurng Cheng

**Affiliations:** 1 Division of Cardiology, Department of Internal Medicine, Shuang Ho Hospital, Ministry of Health and Welfare, Taipei Medical University, New Taipei City 23561, Taiwan; liumdcv@tmu.edu.tw; 2 Division of Cardiology, Department of Internal Medicine, School of Medicine, College of Medicine, Taipei Medical University, Taipei City 11002, Taiwan; 3 Department of Biochemistry, School of Medicine, College of Medicine, China Medical University, Taichung City 404328, Taiwan

Heart failure remains a significant global health concern, profoundly impacting the lives of millions and placing considerable strain on healthcare systems worldwide. Although diagnostic and therapeutic advancements have led to measurable improvements in patient care, substantial challenges persist—underscoring the need for ongoing research and innovation. Emerging strategies such as remote monitoring, telemedicine, and biomarker-based risk stratification have contributed to better clinical outcomes. Yet, critical gaps remain in our understanding of disease progression, treatment responsiveness, and the development of individualized care pathways. This Special Issue brings together a series of contemporary investigations that address key dimensions of heart failure management. Remote monitoring and telemedicine are reshaping the delivery of care [1]. For instance, the ECOST-CRT study demonstrated that remote monitoring in patients undergoing cardiac resynchronization therapy significantly reduced hospitalizations and enhanced device performance [2]. Broader implementation of digital health programs has further highlighted their potential to reduce acute care utilization and improve access to timely interventions [3]. Biomarker-guided approaches are increasingly informing prognosis and therapeutic decisions. Immune markers—such as the neutrophil-to-lymphocyte ratio and the lymphocyte-to-white blood cell count ratio—have emerged as valuable predictors of adverse outcomes [4,5]. Investigations into innate immunity have also shed light on the inflammatory mechanisms that contribute to heart failure pathophysiology [6]. Pharmacological innovations continue to expand treatment options, particularly for patients with preserved ejection fraction and metabolic comorbidities. The FINEARTS-HF trial demonstrated the efficacy of finerenone in slowing disease progression among heart failure patients with obesity [7]. Similarly, findings from the SUMMIT trial underscored tirzepatide’s potential to alleviate circulatory overload and mitigate end-organ damage in obese individuals [8,9]. Lifestyle interventions remain foundational to heart failure management [10]. Dietary modifications have shown promise in lowering lipoprotein(a) levels and reducing overall cardiometabolic risk [11,12]. Remote cardiac rehabilitation programs have emerged as viable alternatives, especially in resource-limited settings, offering improvements in functional capacity and patient adherence [13]. These findings reinforce the importance of integrating behavioral strategies into comprehensive care models. Taken together, the studies featured in this Special Issue advocate for a multidimensional approach to heart failure management—one that combines technological innovation, biomarker refinement, pharmacological advancement, and lifestyle optimization. Nonetheless, several challenges remain, including the need for personalized treatment strategies, enhanced adherence to care protocols, and deeper insights into the immunometabolic underpinnings of heart failure. By synthesizing current evidence and highlighting emerging methodologies, this Special Issue calls for sustained interdisciplinary collaboration and innovation. Bridging existing knowledge gaps and embracing novel approaches will be essential in developing more effective, patient-centered solutions for this pervasive and complex condition.

Recent advances in heart failure management have been driven by technological innovation, refined risk assessment tools, and evolving dietary strategies. These developments have not only enhanced clinical outcomes but also underscored the importance of a cohesive, multidisciplinary approach to this complex condition. Telemedicine and remote monitoring have markedly transformed patient care, enabling earlier detection of physiological changes and facilitating timely clinical interventions. Evidence supports the effectiveness of remote monitoring in improving outcomes by allowing prompt responses to shifts in patient status [1]. Telemedicine, in particular, has broadened access to care for underserved populations, reinforcing the value of sustained investment in digital health infrastructure. Progress in risk stratification has paved the way for more individualized treatment strategies. Biomarkers such as the lymphocyte-to-white blood cell count ratio have emerged as reliable prognostic indicators in heart failure, aiding clinicians in identifying patients at elevated risk [5]. Integrating these markers into routine practice enhances clinical decision-making and supports more targeted therapeutic interventions. Dietary patterns have also gained recognition as key contributors to heart failure prevention and management. The cardioprotective effects of the Mediterranean and DASH diets are well established, particularly in their capacity to reduce heart failure risk [14]. Additionally, dietary modifications that influence lipoprotein(a) levels offer valuable insights into the role of nutrition in cardiovascular care [12]. These findings highlight the critical interplay between diet and heart health, warranting further investigation into nutrition-based strategies as essential components of comprehensive heart failure management.

This Special Issue explores recent advances in heart failure research, emphasizing innovations in diagnostic methodologies, therapeutic strategies, and emerging treatment modalities. Among the promising developments are novel metrics such as the Lipid Accumulation Product (LAP) and Cardiometabolic Index (CMI), which have demonstrated utility in identifying metabolic syndrome risk—even in populations traditionally considered low-risk, such as athletes. These tools offer a pathway toward more personalized and preventive diagnostic frameworks [10]. Nutritional interventions continue to gain prominence in heart failure management. Findings from the OmniHeart trial and related studies underscore the critical link between dietary patterns and metabolic health. In particular, the modulation of lipoprotein(a) levels through tailored nutrition strategies highlights the importance of individualized, lifestyle-based assessments in cardiovascular risk reduction [11,15]. Cardioprotective agents such as grape seed extract (GSE) and L-carnitine have shown encouraging results in mitigating doxorubicin-induced cardiotoxicity. These compounds have been associated with improved histopathological outcomes and preserved cardiac function [16]. GSE’s antioxidant properties appear especially beneficial when combined with exercise, offering synergistic protection against oxidative stress [17,18]. Likewise, L-carnitine has demonstrated potential in pediatric oncology settings, where it may serve as a supportive adjunct during high-dose anthracycline therapy [19]. These findings merit further clinical validation through well-designed trials. Antidiabetic therapies—including sodium–glucose cotransporter-2 (SGLT2) inhibitors and glucagon-like peptide-1 receptor agonists (GLP-1 RAs)—are increasingly recognized for their perioperative benefits in cardiac surgery [20]. Liraglutide, a GLP-1 RA, has shown promise in preclinical models by restoring cardiac function following isoprenaline-induced myocardial injury and preventing heart failure progression [21]. Agents such as empagliflozin and semaglutide have also demonstrated efficacy in enhancing myocardial autophagy and reducing inflammation [22,23]. SGLT2 inhibitors contribute to left ventricular remodeling and reduce perioperative complications [24,25], while semaglutide has been shown to improve cardiac function in patients with heart failure and preserved ejection fraction [25]. Electrocardiographic (ECG) parameters are gaining traction as predictive tools in cardiac resynchronization therapy (CRT). Tanasescu’s work illustrates how specific ECG features correlate with Kansas City Cardiomyopathy Questionnaire (KCCQ) scores, offering a refined approach to CRT optimization [26]. Advances in pacing techniques, including left bundle branch and biventricular pacing, continue to enhance cardiac performance and underscore the value of integrating ECG insights into individualized care planning [27]. Despite these strides, translating research into routine clinical practice remains a challenge. While the Mediterranean and DASH diets have been associated with reduced heart failure risk, long-term, population-based studies evaluating their sustained impact are still limited [14]. The role of diet in influencing cardiovascular mortality and metabolic health among older adults also warrants further investigation across diverse cohorts [28]. Similarly, the safety and efficacy of cardioprotective agents such as GSE and L-carnitine require confirmation through large-scale clinical trials [16,17,18,19]. Implementation barriers—including disparities in healthcare resources, variability in clinical practice, and infrastructural limitations—continue to impede the adoption of evidence-based strategies. Although telemedicine and remote monitoring have expanded access to care in underserved regions, issues such as patient adherence and data reliability remain unresolved [1,29]. Nevertheless, large-scale remote monitoring initiatives have successfully reduced hospitalization rates, reinforcing the need for robust integration and operational frameworks [3]. Looking ahead, interdisciplinary collaboration among cardiologists, nutritionists, pharmacologists, and data scientists will be essential to bridge existing knowledge gaps, harmonize care standards, and accelerate global progress in heart failure research.

Heart failure research continues to evolve, propelled by technological innovation and methodological refinement. This Special Issue highlights emerging trends that are poised to shape the future of heart failure management, with particular emphasis on artificial intelligence (AI), nutritional strategies, biomarker development, and personalized care. AI is rapidly transforming cardiovascular medicine by enhancing predictive modeling and supporting data-driven clinical decisions. AI-enabled platforms have shown efficacy in detecting early complications and stratifying risk through the analysis of large-scale datasets, particularly in remote monitoring contexts [1]. Telemedicine and AI-integrated patient monitoring systems are improving adherence and reducing hospitalizations by capturing symptom-specific biometric data [3,28]. In parallel, AI applications in cardiac rehabilitation and chronic disease management are expanding access to care and optimizing resource utilization [13]. When embedded into clinical workflows, these technologies facilitate timely and informed decision-making, marking a paradigm shift in heart failure care delivery. Despite these advances, several areas warrant further investigation. Long-term adherence to heart-healthy dietary patterns—such as the Mediterranean and DASH diets—and their cumulative effects on heart failure prevention and progression remain underexplored [14]. Given the influence of nutrition on cardiometabolic risk factors, including lipoprotein(a), developing strategies to improve dietary compliance may contribute meaningfully to risk reduction [11,12]. Tailored interventions for older adults and individuals with multiple comorbidities are also essential, as population-specific studies can inform more nuanced clinical approaches [27]. Progress in biomarker research offers promising avenues for refining patient stratification and monitoring. Ratios such as lymphocyte-to-white blood cell count and neutrophil-to-lymphocyte have emerged as potential prognostic indicators [4,5]. Further exploration of immune-related mechanisms—particularly those involving innate and adaptive immunity—may reveal novel therapeutic targets and deepen our understanding of heart failure pathophysiology [6,29]. Addressing the complexities of heart failure with preserved ejection fraction remains a critical priority [30]. This subset of patients presents unique diagnostic and therapeutic challenges, underscoring the need for continued research and innovation.

This Special Issue brings together a diverse collection of studies that advance the field of heart failure research, highlighting the value of a multidisciplinary approach to diagnosis, treatment, and prevention. Through the exploration of emerging innovations—including telemedicine, biomarker refinement, and dietary interventions—these contributions address critical gaps in current knowledge and lay the groundwork for future inquiry. The findings presented offer meaningful insights with the potential to reshape heart failure management and improve patient outcomes. Collectively, they reflect the transformative capacity of collaborative research to drive progress and inform clinical practice. As the burden of heart failure continues to grow, sustained engagement among researchers, clinicians, and policymakers will be essential to overcoming persistent challenges. By building on these advancements, the global scientific community moves closer to a more comprehensive understanding of heart failure and its multifaceted treatment landscape. This Special Issue serves not only as a reflection of current progress but also as a call to action—urging continued innovation and unified efforts in addressing one of the most pervasive conditions in cardiovascular medicine.

## Abbreviations

The following abbreviations are used in this manuscript:

HF	Heart Failure
CRT	Cardiac Resynchronization Therapy
ECG	Electrocardiogram
KCCQ	Kansas City Cardiomyopathy Questionnaire
AI	Artificial Intelligence
SGLT2	Sodium–Glucose Cotransporter-2
GLP-1 RA	Glucagon-Like Peptide-1 Receptor Agonist
GSE	Grape Seed Extract
LAP	Lipid Accumulation Product
CMI	Cardiometabolic Index
DASH	Dietary Approaches to Stop Hypertension
LVEF	Left Ventricular Ejection Fraction
OmniHeart	Optimal Macronutrient Intake Trial for Heart Health
